# Predictors and histological effects of preoperative chemoradiotherapy for rectal cancer and control of lateral lymph node metastasis

**DOI:** 10.1186/s12876-022-02414-7

**Published:** 2022-07-08

**Authors:** Hiroshi Miyakita, Lin Fung Chan, Kazutake Okada, Hajime Kayano, Masaki Mori, Sotaro Sadahiro, Seiichiro Yamamoto

**Affiliations:** Department of Digestive System Surgery, Tokai University, School of Medicine, 143 Shimokasuya Isehara, Kanagawa, 259-1193 Japan

**Keywords:** Chemoradiotherapy, Lateral lymph node, Rectal cancer

## Abstract

**Introduction:**

Standard treatment strategy for low rectal cancer in Japan is different from Western countries. Total mesorectum excision (TME) + lateral lymph node dissection (LLND) is mainly carried out in Japan, whereas neoadjuvant chemoradiotherapy (nCRT) + TME is selected in Western countries. There is no clear definition of preoperative diagnosis of lateral lymph node metastasis. If we can predict lateral lymph node swelling that can be managed by nCRT from lateral lymph node swelling that require surgical resection, clinical benefit is significant. In the current study we assessed characteristics of the lateral lymph node recurrence (LLNR) and LLND that can be managed by nCRT.

**Patients and Methods:**

Patients with low rectal cancer (n = 168) underwent nCRT between 2009 and 2016. We evaluated CEA, neutrophil/lymphocyte ratio (NLR), platelet/lymphocyte ratio (PLR), and lateral lymph node short axis pre and post nCRT, respectively, and also evaluated tumor shrinkage rate, tumor regression grade (TRG). We evaluated the relationship between each and LLNR.

**Results:**

LLND was not carried out all patients. Factors associated with LLNR were PLR and lymph node short axis pre and post nCRT. (p = 0.0269, 0.0278, p < 0.0001, p < 0.0001, respectively). Positive recurrence cut-off values of lateral lymph node short-axis calculated were 11.6 mm pre nCRT and 5.5 mm post nCRT.

**Conclusion:**

Results suggest that PLR before and after CRT was associated with control of LLNR, and LLND should be performed on lateral lymph nodes with short-axis of 5 mm and 11 mm pre and post nCRT.

## Introduction

A standard of care for low rectal cancer is different between Japan and Western countries. While total mesorectum excision (TME) combined with lateral lymph node dissection (LLND) is the golden standard in Japan, [1] neoadjuvant chemoradiotherapy (nCRT) combined with TME is the first choice in Western countries. LLND is usually not carried out in Western countries since lateral lymph node metastasis is considered remote metastasis. [2] Previous studies showed that combined nCRT and LLND for low rectal cancer was effective to reduce local recurrence. [3, 4] and it became a therapeutic option to reduce local recurrence. However, there are no established criteria for LLND indication in which thresholds sizes of lateral lymph node were varied such as 5 or 8 mm in short-axis diameter, or 8 mm in long-axis diameter. [5–9]

There are studies comparing the incidence of lateral lymph node metastases between nCRT + TME + LLND and TME + LLND. Patients who underwent LLND had a positive lymph node metastasis rate of 52.4% and a cut-off value for the size of the lymph node was 8 mm. And the study suggests that the resected lateral lymph node microscopic residual cancer cells were probably destined to disappear if left unresected. [10] Another study showed that tumor regression effect of nCRT increases over time. [11] Previous studies have not shown how much the tissue regression effect of nCRT, which increases over time, can be controlled if metastatic-positive lymph nodes are not removed at surgery. Our group has been carrying out nCRT + TME without LLND as a standard of care, and systemic chemotherapy was applied if the lymph node size increased as postoperatively. It is clinically significant if we can distinguish the lymph nodes which require LLND from the lymph nodes which can avoid LLND by nCRT. Previous studies have not yet assessed factors for LLND indication beside size of the lymph node.

Additionaly, in the present study, we evaluated neutrophil/lymphocyte (NLR), platelet/lymphocyte (PLR), and CEA, which have been reported as predictors of the effects of nCRT. [12–15] Other than that, tumor shrinkage rate, tumor regression grade (TRG) [16], and lateral lymph node short axis are used to determine if they are predictors of lateral lymph node recurrence (LLNR).

## Subjects and methods

### Patients

Between 2009 and 2016, 168 patients with low rectal cancer (cT3-4, N any) whose anal verge (AV) was 7 cm and less and underwent nCRT (40-45 Gy) with oral Uracil/Tegafur or S-1 chemotherapy. TME was carried out at 6 to 8 weeks after nCRT, and LLND was not performed.

### Assessment of therapeutic effect

As predictive factors of nCRT outcome, CEA levels, NLR, PLR prior to and after the nCRT were assessed. As factors of therapeutic effect, TRG and lateral lymph node short-axis diameter were assessed. For CEA, we used 5 ng/mL as our institutional cut off value (COV). Both NLR and PLR were measured from blood samples prior to and after the CRT. We determined COV from the receiver operating characteristic curve (ROC) in which recurrence was positive. Tumor shrinkage rate was calculated by T2-weighted MRI images prior to and after the CRT according to the formula: Tumor shrinkage rate (%) = 100 x [(pre-CRT tumor volume) – (post-CRT tumor volume)] / (pre-CRT tumor volume)]. [17]

Histological assessment was carried out according to the tumor regression grade (TRG) in which grade 1 as complete regression; grade 2 as presence of rare residual cancer cells; grade 3 as increased number of residual cancer cells; grade 4 as residual cancer outgrowing fibrosis, and grade 5 as absence of regression change. [16] Responder was defined as TRGs 1 and 2.

Lateral lymph node short-axis diameter was measured using MRI and CT images. Cancer recurrence was diagnosed by CT, and only initial recurrence lesion was evaluated to rule out lateral lymph node metastases from local recurrence.

### Statistical analysis

The COV for each score was calculated by risk evaluation analysis, performed using ROC in which the recurrence of LLND was considered as a positive result. The patients were divided into 2 groups according to whether their score was less than the COV or equal to or greater than the COV, and the incidence of recurrence was compared. Lateral lymph node recurrence rate was assessed by Kaplan–Meier analysis and multiple logistic regression analysis. We defined p < 0.05 as statistical significance. All statistical analyses were carried out using MP®10 software (SAS Institute Inc., Cary, NC, USA).

This study was approved by the institutional review board of Tokai University (21R167).

## Results

### Patient characteristics

Table 1 shows patient characteristics. Among168 patients, 124 patients were male and 44 patients were female. Based on ypT staging, 26 patients displayed pCR (15.4%). The TRGs 1 and 2 were 65 patients, and TRGs 3 and 4 were 103 patients. Local recurrence was found in 28 patients (16.6%) and the lateral lymph node recurrence was found in 5 cases (3%). Median observational period was 1,874 days.

**Table1:** Patient’s Characteristic

Sex	Male	124	TRG‡	1–2	65
	Female	44		3–4	103
age	Range	33–81	Adjuvant Chemotherapy	no	106
	Median	62		5-FU	58
cT Stage	3	129		5-FU + OX	4
	4a	30	Local recurrence	Yes	28
	4b	9		No	140
cN Stage	-	65	Lateral lymph node recurrence	Yes	5
	+	103		No	163
ypT Stage	0	7	Distant recurrence	Yes	43
	1a/b	9		No	125
	2	40	Procedure	LAR§	119
	3	85		APR	42
	4a/b	1		Hartmann	3
	pCR†	26		Local Excision	4
ypN Stage	-	121			
	+	44			
	X	3			

*pCR* Pathological complete response, *TRG* Tumor regression grade, *LAR* Low anterior resection, *APR* Abdominoperioperineal resection

### Association between predictive factors of CRT effect and lateral lymph node recurrence

**Fig. 1:**
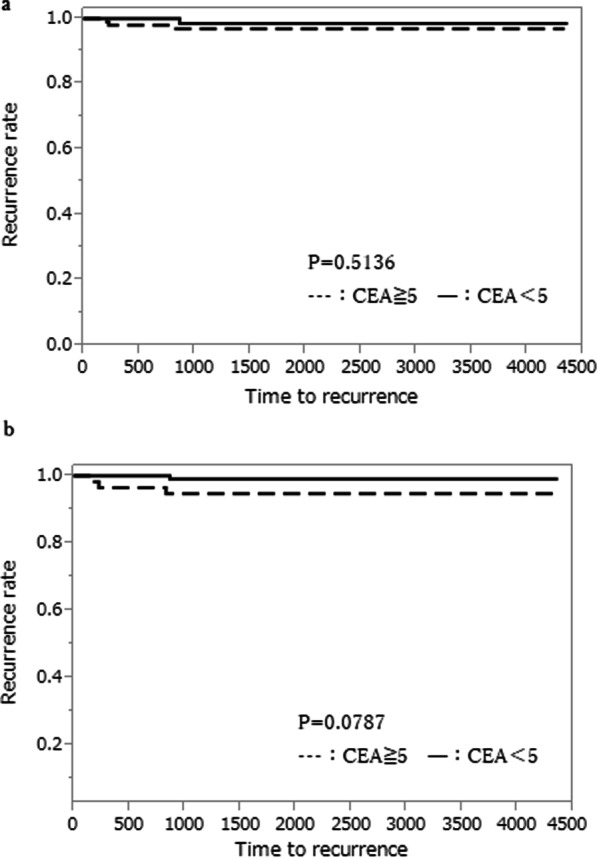
Association between predictive factors of CEA level and lateral lymph node recurrence. **a** Association between predictive factors of Pre-CRT CEA level and lateral lymph node recurrence. **b** Association between predictive factors of Post-CRT CEA level and lateral lymph node recurrence

**Fig. 2:**
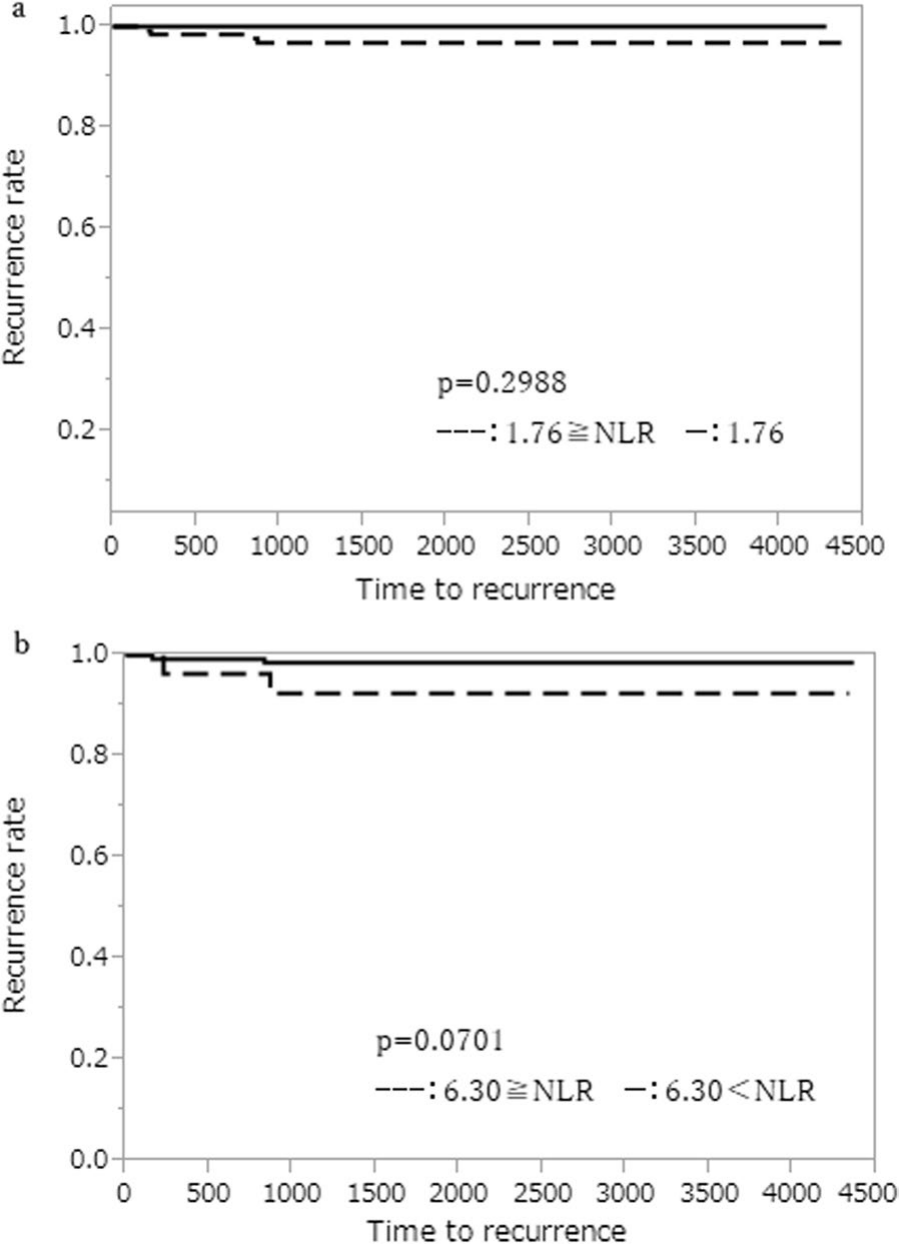
Association between predictive factors of NLR and lateral lymph node recurrence. **a** Association between predictive factors of Pre-CRT NLR and lateral lymph node recurrence. **b** Association between predictive factors of Post-CRT NLR and lateral lymph node recurrence

**Fig. 3:**
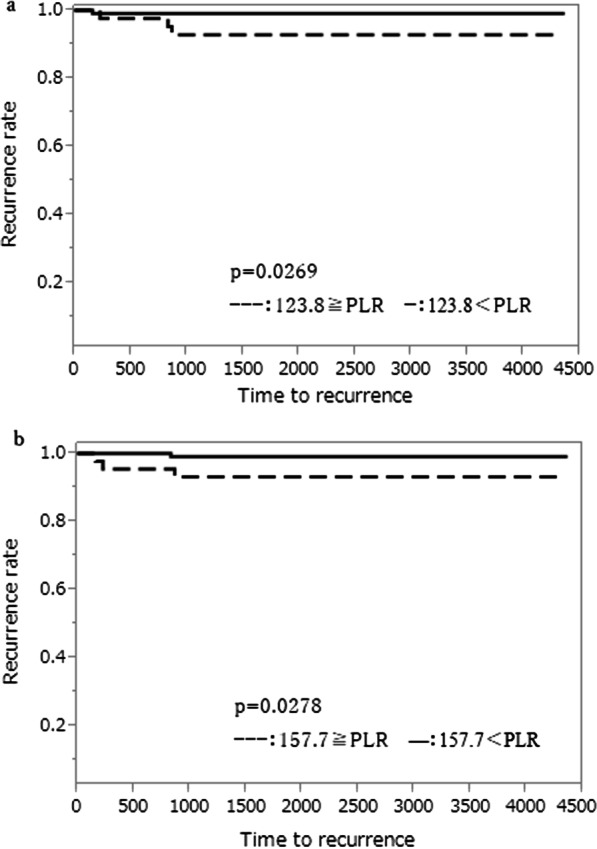
Association between predictive factors of PLR and lateral lymph node recurrence. **a** Association between predictive factors of Pre-CRT PLR and lateral lymph node recurrence. **b** Association between predictive factors of Post-CRT PLR and lateral lymph node recurrence

Pre- and post-CRT CEA levels were not statistically related with lateral lymph node recurrence (p = 0.5136, p = 0.0787, respectively, Fig. 1a, b). The COVs of NLR were 1.76 at pre-CRT and 6.30 at post-CRT, respectively. There were no statistical relations between COVs at pre- and post-CRT and lateral lymph node recurrence (p = 0.2988, p = 0.0701, Fig. 2a, b). The COVs of PLR were 123.8 at pre-CRT and 157.7 at post-CRT, respectively. There were significant statistical relations between pre- and post-CRT PLR and lateral lymph node recurrence. The PLR at COV and above significantly increased in patients with lateral lymph node recurrence (p = 0.0269, p = 0.0278, Fig. 3a, b).

### Association between tumor shrinkage rate, histological effect, lateral lymph node short-axis diameter with CRT, and the lateral lymph node recurrence

**Fig. 4:**
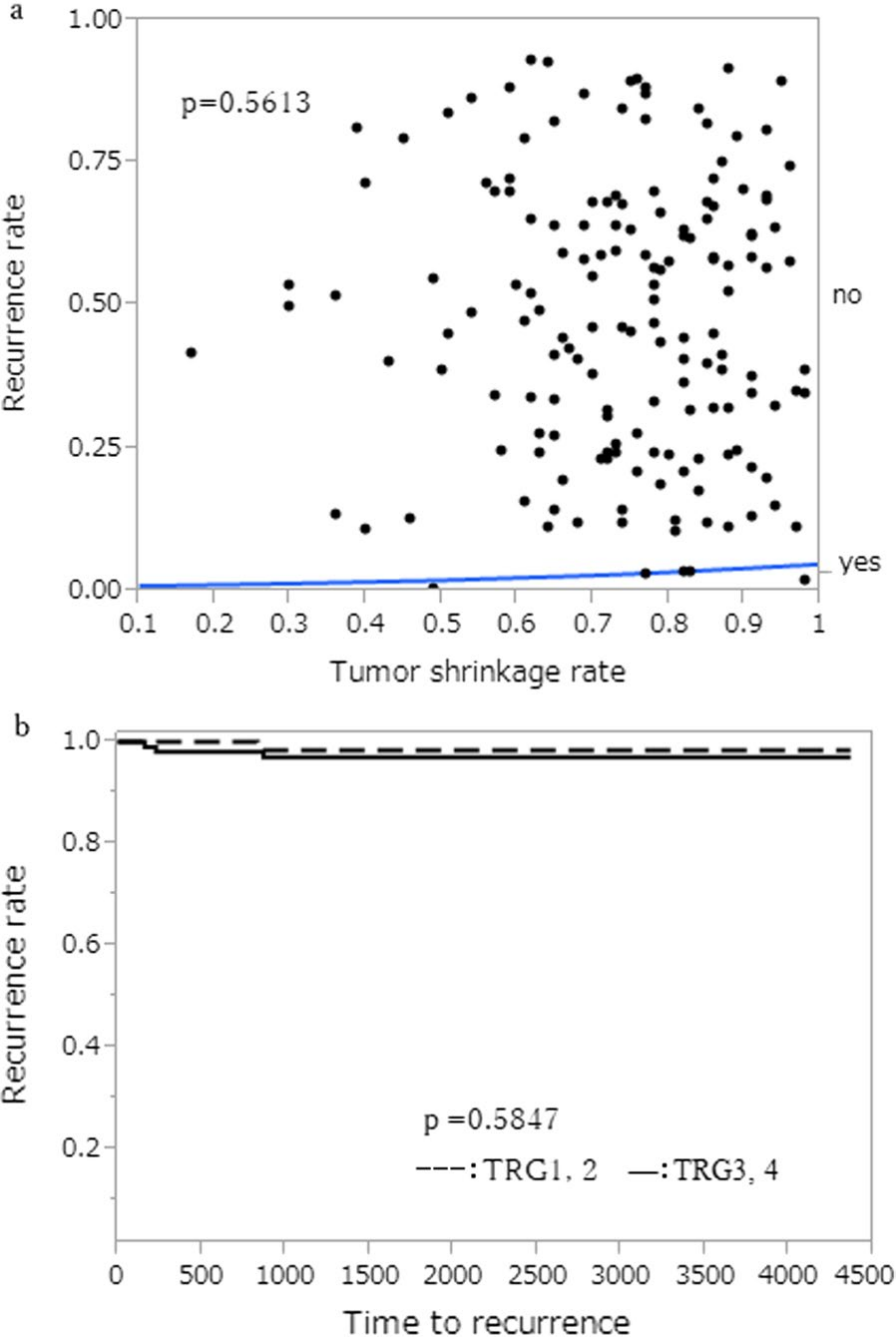
Association between tumor shrinkage rate, histological effect of CRT, and the lateral lymph node recurrence. **a** Association between tumor shrinkage rate and the lateral lymph node recurrence. **b** Association between histological effect of CRT, and the lateral lymph node recurrence

Median value of tumor shrinkage rate was 77.0%. There was no statistical relation between tumor regression rate and lateral lymph node recurrence (p = 0.5613, Fig. 4a). The TRGs 1 and 2 were 65 patients (%) and the TRGs 3 and 4 were 103 patients. There were no statistical relations between TRGs and lateral lymph node recurrence (p = 0.5847, Fig. 4b).

**Fig. 5:**
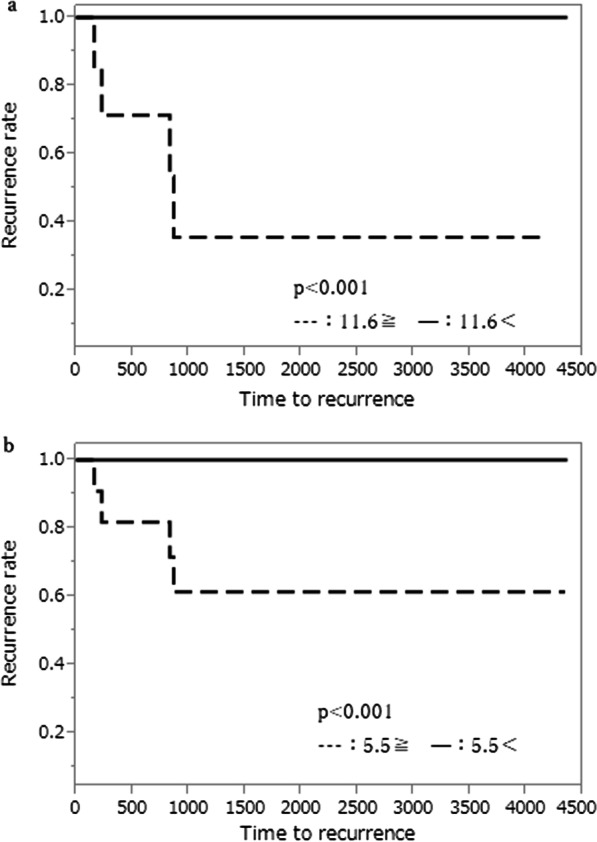
Association between lateral lymph node short-axis diameter of CRT, and the lateral lymph node recurrence. **a** Association between lateral lymph node short-axis diameter of pre- CRT, and the lateral lymph node recurrence. **b** Association between lateral lymph node short-axis diameter of post- CRT, and the lateral lymph node recurrence

The COV of lateral lymph node short-axis diameter was 11.6 mm at pre-CRT and 5.5 mm at post-CRT, respectively. There were 7 patients whose pre-CRT diameters were 11.6 mm or larger (4.2%). Of those, five patients developed lateral lymph node recurrence, and the recurrence rate was 71.4%. No patients whose pre-CRT lymph node diameters less than 11.6 mm developed the lateral lymph node recurrence. There were 11 patients (6.5%) whose post-CRT lateral lymph node short-axis diameters were 5.5 mm and larger. Of those, five patients developed lateral lymph node recurrence, and the recurrence rate was 45.5%. There was a statistically significant relations between pre- or post-CRT and lateral lymph node short-axis diameter (p < 0.001, p < 0.001, respectively, Fig. 5a, b).

## Discussion

Our study suggests that the histological effect of rectal lesions by CRT does not support the omission of LLND. And the minor axis of the lateral lymph node and PLR are risk factors for recurrence of the lateral lymph node.

The American Society of Colon and Rectal Surgeons (ASCRS) strongly recommends not performing LLND in the absence of swollen lateral lymph nodes. However, it does not mention appropriate treatment strategy for patients with swollen lateral lymph nodes. [18]

Fujita et al. reported that 5-year relapse-free survival rate in the TME + LLND group wan not improved compared with TME group. Morevoer, lateral pelvis recurrence was significantly high in TME group and, recurrence of lateral lymph nodes has been reported to be the leading cause of pelvic recurrence in the absence of LLND after CRT, [19, 20] and the usefulness of LLND has also been recognized in Western countries.

Hazen et al. reviewed the treatment of lateral lymph nodes in rectal cancer and state that they will change towards selective LLND in the future. [21] Therefore, it is important to develop predictors of lateral lymph node metastasis.

Regarding lymph node size and cancer recurrence, previous studies investigated LLND indication by lymph node size, and specific criteria have not yet been established. Kawai et al. reported that lateral lymph node recurrence rate was 1.6% in post-CRT + TME and TME + LLND was performed on patients with 8 mm LLN before surgery, and the positive rate for lateral lymph node metastasis was 52.4%. And the estimated incidence of LLN metastases after nCRT was 9.3%. [10]

They also reported that some of patients who underwent LLND did not develop local cancer recurrence through post-operative observation and assessment of lymph node size had 1-mm interobserver error, and there is a limit to the evaluation based on the lymph node size alone. [10] In the currency study, patients with pre-CRT lymph node minor-axis diameter less than 11.6 mm and post-CRT diameter less than 5.5 mm did not develop lateral lymph node recurrence. The short-axis is an easy-to-use index for clinical management, and we suggest to perform LLND in case of increased risk according to size. Also, we believe that nCRT can eliminate bilateral or unilateral LLND and can provide shorter surgery in some patients. Whether it would really decrease local recurrence or not can be answered by a prospective study.

While the results in the current study did not show significant difference compared to previous studies, only 5 patients developed lateral lymph node recurrence. Further studies with additional cases are necessary.

### Predictive factors of therapeutic effect, histological effect, and lateral lymph node recurrence

Previous studies showed that patients with good histological effect had favorable outcome. [22, 23] However, some patients with TRG 1 exhibited lymph node metastasis.

It has been reported that 4.6% of patients with TRG1 were positive for lymph nodes in the mesorectum, and that the lymph nodes in the mesorectum were associated with the TRG of the tumor itself. [24, 25] However, its association with lateral lymph nodes has not been investigated. Although it is unclear why there is a difference in the control of lymph nodes in the irradiation range, our study found no association between lateral lymph node recurrence, TRG, and tumor shrinkage.

Inflammation is associated with tumor formation, and various predictive factors are investigated. Previous studies showed that CEA, NLR, and PLR were related to the histological effect. [12–15] Therefore, in the present study, we assessed the relations between each factor and the lateral lymph node recurrence, and found significant relations between pre- and post-CRT PLRs and lateral lymph node recurrence. Lee et al. reported PLR had a close relation with pathological complete response (pCR). [14] Current results and previous report suggest that PLR is useful not only for assessment of histological effect, but also it can be used as a predictive factor of lateral lymph node recurrence.

While PLR can be easily calculated from standard blood sampling, there is no consensus regarding COV. Therefore, it is difficult to use for clinical management.

We are aware of several limitations in the current study, which include a single institutional study and small sample size. In the current study, positive cases were defined as lateral lymph node recurrence without local recurrence, however, true lateral lymph node recurrence might not be captured. Despite these study limitations, the current study is the first study which assessed lateral lymph node recurrence by various factors not limited to the lymph node size. Further studies with additional cases are necessary.

## Conclusion

The current study showed that patients whose lymph node short-axis diameters less than 11.6 mm at pre-CRT and 5.5 mm at post-CRT did not develop lateral lymph node recurrence. There was no relation between histological effect and lateral lymph node recurrence. Results suggest that pre- and post-CRT PLRs may be a useful predictive factor for management of lateral lymph node recurrence in patients with rectal cancer. It was suggested that it may help in choosing a surgical procedure.

## Data Availability

The approval to access the collected data used in this current study is limited by Tokai University, and it is not allowed to distribute or make patient data directly available to other parties. The datasets generated and/or analyzed during the current study are not publicly available due to limit by Tokai university and do not include allowance to forward or share data with other researchers but are available from the Hiroshi Miyakita on reasonable request.
